# “Why screen if we cannot follow-up and manage?” Challenges for gestational diabetes screening and management in low and lower-middle income countries: results of a cross-sectional survey

**DOI:** 10.1186/s12884-016-1143-1

**Published:** 2016-11-08

**Authors:** Bettina Utz, Vincent De Brouwere

**Affiliations:** Public Health Department, Institute of Tropical Medicine (ITM), Nationalestraat 155, 2000 Antwerp, Belgium

**Keywords:** Gestational diabetes mellitus, Maternal health, Maternal morbidity, Neonatal health, Non-communicable diseases, Health systems, Low and lower-middle income countries

## Abstract

**Background:**

The prevalence of gestational diabetes (GDM) in low and lower middle income countries (LLMIC) is increasing. Despite its associated short and long term complications for mothers and their newborns, there is a lack of knowledge about how to detect and manage GDM. The objective of our study was to identify the challenges that first line healthcare providers in LLMIC face in screening and management of GDM.

**Methods:**

We conducted a cross-sectional survey of key informants from 40 low and lower-middle income countries in Africa, South-Asia and Latin-America by sending out questionnaires to 182 gynecologists, endocrinologists and medical doctors. Sixty-seven respondents from 26 LLMIC provided information on the challenges they encounter. Data was thematically analyzed and revealed eight overarching themes, including guidelines; human resources; access; costs; availability of services, equipment and drugs; patient and community factors; and collaboration and communication.

**Results:**

Unavailability of guidelines combined with lack of knowledge about GDM on the part of both providers and patients poses a substantial barrier to detection and management of GDM, leading to deficiencies in screening and counseling. Limited access to regular monitoring and follow-up care as a result of distance and costs, in particular with respect to additional expenses related to specific tests and changes in diet were identified as important challenges. Services were not available at all levels nor was adequate testing equipment. Patient factors included lack of motivation and compliance with the recommended therapy. Respondents also highlighted the lack of communication and collaboration between different specialists and treatment delays as a result of patients being seen by multiple providers.

**Conclusions:**

Providers from LLMIC face various challenges related to screening and managing GDM. Policy makers need to address these challenges by strengthening their health care system as a whole and by assuring that non-communicable diseases are better integrated into the existing packages of free or subsidized maternal health care.

## Background

Global prevalence of diabetes mellitus in women of reproductive age is steadily increasing [1]. Similarly, the incidence of hyperglycemia diagnosed for the first time during pregnancy is on the rise and has reached nearly 17 % worldwide [1, 2]. The proportion of women with gestational diabetes (GDM) has not yet been assessed in many low and lower-middle income countries (LLMIC). However, the few studies conducted in Sub-Saharan Africa reveal prevalence rates in pregnant women ranging from 2 % to 14 % in Sub-Saharan Africa [3–5], and up to 18 % in South Asia [6, 7].

These numbers are worrying, as GDM is associated with a range of immediate and long term complications for both mothers and their newborns. Babies of affected mothers are at higher risk of being delivered preterm, of being macrosomic or suffering from hypoglycemia, jaundice or respiratory distress. Mothers have an increased risk of developing pregnancy-induced hypertension or pre-eclampsia, of experiencing a shoulder dystocia or a postpartum hemorrhage and are more likely to deliver by caesarean section [8]. Maternal diabetes is also a known condition associated with stillbirths [9], 10 % of which are attributable to non-communicable diseases [10]. In the long term, GDM affected women and their newborns are more prone to developing manifest diabetes mellitus [8, 11].

Although guidelines from high income settings on GDM screening and management are available and freely downloadable from the internet [12, 13], there is a lack of knowledge about the extent to which guidelines developed in high income settings are appropriate for use in less developed countries, where resources are scarce and access to care is limited [14].

The objective of our survey was therefore to identify existing challenges for first line health care providers in LLMIC regarding the detection and management of GDM and to use the findings to inform leaders of professional associations and policy makers involved in maternal and newborn health programmes. By identifying these challenges, the study aimed to contribute to the development of strategies for improving detection and management of GDM.

## Methods

**Fig. 1:**
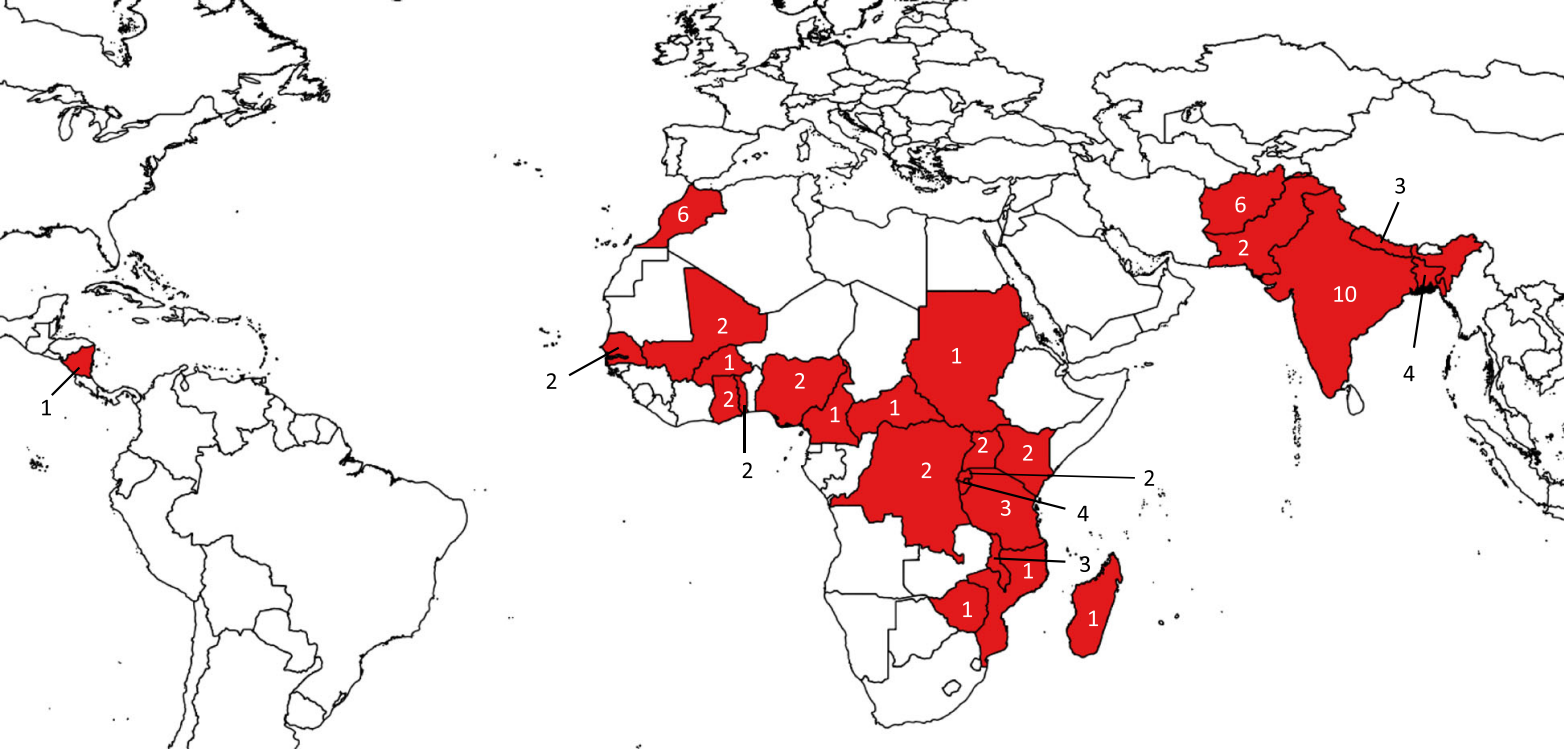
Map depicting location of respondents

We conducted a cross-sectional survey in 40 out of the 56 LLMIC in Africa, South-Asia and Latin- America eligible for World Bank funding [15] to assess clinical practices used in detection and management of GDM and to explore existing challenges. To identify key informants, such as representatives of national professional societies or leading gynecologists in low and lower middle income settings we contacted members of our institutional networks in the respective countries. Identified key contacts were invited to participate in the survey. We then used snowball sampling, asking these key contacts to identify additional health care professionals in their respective countries, including gynecologists, endocrinologists and medical doctors working in the public and/or private sector at all levels of the health care system and involved in providing care to pregnant women and in screening and management of GDM. The survey tool, a self-administered semi-structured questionnaire containing a mixture of multiple choice and open-ended questions, was available in four languages (English, French, Spanish or Portuguese) and was sent out by email to a total of 182 gynecologists, endocrinologists and medical doctors in 40 LLMIC. Questionnaires were not sent to countries that were either affected by crisis or war (5) or where we failed to identify a key informant (11). Returning the completed questionnaire was considered as consent to participate in the study. A total of 77 respondents from 27 LLMIC returned the questionnaires and 67 respondents from 26 countries answered the open ended questions on challenges they encountered with GDM screening and management (Fig. 1). All data was handled anonymously with profession and country as sole identifiers and double entered into SPSS Version 21. All open responses were copied into an excel file and thematically analyzed. After sorting and grouping the data, eight thematic areas were identified. These included guidelines, human resources, access, costs, availability of services, of equipment and drugs, patient and community factors as well as collaboration and communication. Ethical approval for the study was granted by the Institutional Review Board of the Institute of Tropical Medicine, Antwerp, Belgium.

## Results

Various challenges regarding GDM detection and management were highlighted by 67 survey respondents, including 59 gynecologists, five endocrinologists and three general practitioners from 26 LLMIC. 61.2 % of the respondents providing information on challenges were from Africa, 37.3 % from South-Asia and 1.5 % from Latin-America. 22.4 % of them were representatives at national level, 49.3 % were working in the public sector, 23.9 % in private facilities (including faith-based and non-governmental) and 4.5 % in both the public and private sector.

### Guidelines

Several respondents highlighted problems concerning guidelines in their countries. Some informants complained that no standard protocols were available in their settings. In countries where protocols were available, these were sometimes not adequately disseminated, not easily comprehensible or not used by the health care providers (Table 1a).

**Table 1:** Reported challenges with GDM screening and management grouped by thematic area

a. Guidelines	b. Human resources	c. Access & follow-up	d. Costs	e. Service availability	f. Equipment & drugs	g. Community & patients	h. Collaboration & coordination
Lack of national guidelines/standard protocols	Not enough awareness of problem as no prevalence studies/poor knowledge about GDM	Access (travel)/access to care	Patients have to pay, not affordable to screen and manage/socioeconomic status does not allow to test all women, even those with high risk	No government facility for screening/screening not in all government facilities	No screening reagents/limited resources to test/to have sufficient lab supplies for measurements of GDM	Lack of knowledge in population/low community information/low patient awareness/lack of knowledge of women about diabetes risk and its frequency	No multidisciplinary team with endocrinologist and nutritionist
Limited guidelines for screening/management	Lack of provider sensitization	15 % women attend public facilities only	Hard for poor to get repeated test done and hard to convince them	GDM only screened and managed at tertiary and private level	Stock-out of screening material and test strips/stock out lab equipment and reagents	Ignorance and false belief about diabetes development/ignorance about diabetes and its consequences in pregnancy/	Multidisciplinary collaboration challenge because few endocrinologists and neonatologists
Guidelines not properly disseminated and used	Lack of capacity/technical competency/no capacity building/training/few lab technicians know to do O’Sullivan test	ANC coverage low	Costs of lab tests and ultrasound/costs of treatment and tests/costs for travel, investigations, medication, hospitalization	Laboratory not available 24/7	Unavailability of test strips and sometimes glucometers	Get patient to understand complications of diabetes/spending a lot of time convincing them about long term problems and also have to ring and remind about follow-up visits	Pregnancy not managed together by endocrinologist and obstetrician
Policy guidelines not well articulated	Most providers screen in case of risk factors only/screening not even done when risk factors present or when previous obstetric history would indicate	Late presentation for ANC/late booking/emergency deliveries, women were not follow-up before	No insurance coverage for most, so cannot afford cost of follow-up	Shortage of laboratories in the public sector/good labs only urban	Sometimes glucose strips not available/screening not routinely as no dipsticks	Low compliance to ANC and testing/in first trimester not able to do test in patient with vomiting/some patients easily nauseated and may throw up after anhydrous glucose/2nd and 3rd trimester: some patients are not willing to wait for two hours	Laboratory service not fast and patient has to wait for result
Limited access to internet	Inadequate counselling/If proper counselling mothers very cooperative/no educated midwives who can counsel women in community	Patient without fridge needs to go to close center for treatment but with the problem of transport	Long hospitalization to balance glucose levels/patients requested to get glucometer even while admitted	No screening because of technical problems of lab	Many difficulties in the lab to do correctly O’Sullivan test and HbA1C, primarily due to lack of reagents and organizational issues	Frequent pricking and venous puncture/SMBG cumbersome/difficulty of self-monitoring blood glucose/many patients illiterate and cannot use rapid test/rare that women have a private glucometer	Result seen after 1 week by doctor/late detection by gynecologists
No forum for updates	Few specialists interested in GDM/not enough endocrinologists/nutritionist may not be around/no nutritionists	Majority of pregnant women not followed-up correctly/poor monitoring of patients	Patients have to pay for additional exams and drugs: therefore many women are not screened-treated/financial access limit the demand for specific tests	Glycaemia not regularly checked at lower level of care	Glucose availability/getting anhydrous glucose for tests	Some patients do not undergo screening at recommended times/patient does not come at advised delivery date/patients only come when complication	Patient has to consult several doctors for management
		Surveillance problem (glycaemia, lab, US, CTG) to best plan delivery	OGTT requested at clinic and patients do it at lab when they have money for it/O’Sullivan test too expensive and often not done in public labs	No routine blood tests	Calibration of glucometers not checked against lab standards	Patients don’t always follow management instructions/non-compliance with investigations, diet control/deficient compliance, needs husband cooperation, convince for prevention	Crowding in maternity does not allow to screen routinely and follow-up
		Women lost to follow up/Mothers are not returning for postnatal follow-up	Ultrasound often expensive and done in private facilities	No general screening so most cases are picked up late	Difficult to get charts for glucose surveillance	GDM complex, needs close supervision while patient has domestic commitments/no gym, only brisk walking as exercise possible	Two hours blood sample needs to be taken to lab in time otherwise result error/organization for OGTT is a challenge
		Glucose not well monitored after discharge	Costs for medication affect patient compliance/difficulties to buy insulin/expensive glucose strips for home monitoring	Non availability of O’ Sullivan test and OGTT/for OGTT patient sent to secondary level/O’Sullivan test often not done in public labs	Shortage of drugs to treat GDM in the public sector	Adherence to nutrition/adherence to diet/discontinuation of diet, exercise and drugs/Inadequate control of glucose by diet and exercise (lack of motivation)/getting patients to follow diet and to take insulin if required/difficulty for women to keep their sugar level balanced/very difficult to commence a diet, difficult to change dietary habits	Patients don’t know where to go for screening and follow-up/complex care system for women
			Low income makes diet control difficult as proteins expensive, they eat much carbs/cannot adhere to diet because expensive	Ultrasound often done in private facilities- in the public sector difficult to get appointment	Use of oral agents not very popular with most health care providers	Glucose not well monitored after discharge/getting them to continue physical exercise and diet even after delivery/screening after delivery is systematically prescribed but not always realized by the patient/mothers not returning for postnatal follow-up	
			No financial resources for training of providers	HBA1C hardly performed/Cannot check surfactant levels	Accessibility of insulin analogues/insulin during labor as no infusion pumps	Some fear about insulin/side effects of insulin/women reluctant or irregular to take insulin/difficult to store insulin	

### Human resources

Regarding health care providers’ knowledge, survey respondents indicated that providers lack the required knowledge to screen for GDM and/or manage affected patients. In many settings, no particular training in GDM is provided, partly due to a lack of financial resources. Providers complained that updates are not available, which, coupled with limited internet access, restricts their ability to obtain latest practice updates. Many providers also indicated that general awareness of GDM is low due to lack of knowledge regarding GDM prevalence in their countries. One respondent stated that interest in GDM is limited, even among specialists.

Respondents also mentioned that counselling by providers is inadequate. One informant highlighted a lack of midwifes trained to provide counselling on GDM risks in the communities.

Respondents stated that despite the call for universal screening, some health care providers only screen women with risk factors. Others do not even screen when risk factors are present. Furthermore, is was reported that few lab technicians know about specific tests to detect GDM, such as the O’Sullivan glucose challenge test (Table 1b).

### Access

Access to care was mentioned as a challenge for screening and management. In countries, in which antenatal care (ANC) coverage is low, some patients attend ANC either late in pregnancy or deliver without having attended any ANC consultation. In some settings, only 15 % of women attend public facilities. According to one respondent this can pose problems, particularly because harmonization of practices with the often unregulated private sector is challenging.

Transport can be an obstacle, especially for women receiving insulin. Difficulties in ensuring regular monitoring and follow-up of patients were highlighted by various respondents as major challenges. The complexity of the care system and the need to consult different health care providers also makes access for pregnant women difficult (Table 1c).

### Costs

Financial access to care and the ability to pay for transport and treatment were identified as major barriers to adequate management of GDM. Costs are not only related to accessing health care facilities, but include expenses for specific screening tests, for repeat testing and for other investigations such as obstetric ultrasound scans. Patients diagnosed with GDM often face additional costs of purchasing testing equipment such as glucometers and testing-strips for self-monitoring their blood glucose, as well as for medication and hospitalization. Respondents highlighted that some examinations, such as the O’Sullivan test or obstetric ultrasound scans, are only performed in private laboratories or practices. In addition, costs related to diet play an equally important role. In many low resource settings women are accustomed to a traditional carbohydrate rich diet. Proteins, fruits and vegetables, all ingredients of a balanced carbohydrate restricted diet, are expensive and therefore adhering to such a diet is difficult, especially for poorer women with GDM (Table 1d).

### Availability

#### Services

Service availability plays an important role in the detection and management of GDM. In addition to limited availability of screening services in public facilities of different levels, informants indicated that laboratories often pose a challenge. Technical problems were mentioned, as well as a shortage of laboratories in the public sector. Respondents stated that laboratories do not provide services 24/7 and certain tests, such as the O’Sullivan test, the oral glucose tolerance test (OGTT) and the glycosylated hemoglobin analysis (HbA1C) are not always done. One respondent mentioned that laboratories performing such specific tests are often located in urban areas. Due to difficulties getting these tests done in the public sector, women have to turn to private facilities. Furthermore, there is a shortage of specific health care providers, such as endocrinologists and nutritionists, to provide expert advice in many low income settings (Table 1e).

#### Equipment and drugs

When it comes to equipment, a shortage of laboratory supplies and screening reagents including anhydrous glucose, urine dipsticks and capillary test-strips was mentioned. The regular calibration of glucometers against laboratory standards was considered a technical challenge for ensuring adequate management.

Drug availability was identified as yet another constraint, particularly availability of insulin analogues. Difficulties for patients buying insulin (market availability as well as costs), and challenges for health care providers administering insulin during labour without having infusion pumps at their disposal, were also identified as obstacles (Table 1f).

### Patient/community factors

Survey respondents indicated that patient and community related factors are key barriers. They reported a lack of awareness of diabetes in pregnancy, ignorance of the potential risks and false perceptions regarding GDM. Convincing patients of the need to commence and adhere to a specific diet, to check glucose levels regularly and to attend regular follow-up visits were listed as substantial challenges for health care providers. These challenges were compounded in settings in which the husband’s approval is essential to women’s ability to seek healthcare. Furthermore, adherence to a diet, treatment and regular monitoring may interfere with the domestic responsibilities of affected women.

Discontinuation of diet and treatment were also mentioned and partly explained by lack of motivation. Respondents reported a reluctance to comply with insulin management due to fears related to the administration of insulin and to its side effects. Regarding physical exercise, limited options and the unavailability of dedicated locations (e.g. gyms) where women can exercise often restrict recommendations to brisk walking. Self-monitoring of blood glucose was pointed out as a challenge by several respondents. Illiteracy and the difficulty of acquiring a private glucometer were considered reasons for women’s inability to self-monitor glucose.

Women often do not comply with the recommended diet during pregnancy and discontinue glucose monitoring, their diet and exercise after delivery. Many women would not return for a re-test post-partum, despite having been advised to do so. Other reported patient related factors regarding screening include vomiting after glucose ingestion, particularly when given in the first trimester. It was also highlighted that some women are not willing to wait for two hours to get their blood tested (Table 1g).

### Collaboration and coordination

Inadequate collaboration between different specialists might negatively impact efficient management of GDM, particularly in settings in which additional logistical hurdles exist. Respondents mentioned that collaboration between different professionals is often difficult. Similarly, the lack of multidisciplinary teams was reported. Good coordination is essential, as patients have to circulate between different services and might even have to consult various specialists. Informants mentioned delays in care because patients have to undergo requested tests at the laboratory and then have to revisit the doctor with the results. In case of delays during this process, women are subject to extensive waiting times and this might increase the risk of drop outs. Furthermore, time lags between taking blood and its final analysis in the laboratory could cause measurement errors. Sometimes services are overcrowded, which negatively impacts on health care providers’ capacity to screen patients. Similarly, lack of sufficient patient information in terms of where to go for screening and follow-up might cause further delays and result in loss to follow-up (Table 1h).

### Limitations

The collected challenges present individual views of key informants, mainly of obstetricians from the different countries. As such, these challenges are indicative of problems in individual health care services and might therefore not be generalizable. However, the similarity of the challenges reported by various key respondents would suggest that comparable problems are encountered in several low and lower-middle income countries. The fact that we only collected information from health care providers and did not include the views of patients represents another limitation of this study.

## Discussion

Our study reveals that providers in LLMIC face various challenges related to screening and management of GDM. General availability of guidelines and provision of specific training on GDM is one of the repeatedly mentioned obstacles. Some of these problems have been presented in a recent study conducted in Tanzania, where guidelines on diabetes were only available in 13 % of all facilities and only a third of doctors felt comfortable with the management of diabetes [16].

According to our findings, costs associated with GDM screening and management represent one of the major reasons for which GDM is not adequately addressed in LLMIC. Financial constraints have also been highlighted in a study on diabetes care in Tunisia, where costs related to travel, medication and testing presented serious constraints [17]. Costs of detection and management of GDM are not necessarily part of the packages of care that are offered to pregnant women and these often exclude specific laboratory tests, additional ultrasound investigations or equipment for self-testing such as glucometers and test strips. Furthermore, expenses related to hospitalization in LLMIC have shown to have detrimental effects on families, as these not only involve direct but also substantial indirect costs [18].

However, costs are only one of many factors that hamper adequate management of GDM in LLMIC. Even if costs could be reduced, issues such as compliance and motivation of patients to follow recommendations would persist. Studies have shown that patients who are involved in decision making have better health outcomes [19]. But this would require that patients are informed about their condition. Our findings reveal a lack of knowledge about GDM and its associated risks, as well as false beliefs that may threaten the benefits that can be expected from screening [20]. Good communication between patients and providers, as well as between providers themselves, could ensure that patients feel well informed and, as a consequence, comply with medical advice.

The role of community health workers in diabetes care is not a new concept and their potentially beneficial role in coaching can result in improved compliance and better outcomes [21, 22]. Nevertheless, their role in providing supportive care for GDM affected patients remains to be assessed. To reduce costs and improve monitoring, some innovative ideas have been reported from Kenya, where former GDM patients trained women in self-monitoring of their blood glucose levels and healthcare providers offered backup management support in the form of weekly mobile telephone follow-ups [23].

Several challenges highlighted in our study are related to resources both in terms of staff requirements and regarding equipment and drugs. Solving these problems would require improved overall health care organization that better integrates non-communicable diseases into the existing packages of care. In many LLMIC, maternal and perinatal mortality is still high and there is a persisting need to improve access to and provision of emergency obstetric and neonatal care. However, the parallel emergence of non-communicable diseases affecting maternal and newborn health in these settings will gradually place a double burden on existing health systems [24]. To increase awareness and improve universal detection of GDM in pregnancy, delegation of tasks to mid-or lower level health care providers could represent one potential solution and has already been initiated in India where, according to the latest national guidelines GDM screening now takes place at the first level of care [25]. Required health system related adjustments of GDM detection and management practices target clinical information systems, decision support through evidence based guidelines, redesign of health care delivery systems, self-management support and the use of community resources and are in line with the elements reflected in the chronic care model conceptualized by Wagner [26].

The long list of challenges regarding patient and community related factors mentioned by care givers indicates a need for critical reflection on the reasons for which health care providers face such difficulties in managing patients with GDM in their settings. Are we using patient related obstacles as a scapegoat for our failure to provide adequate care and follow-up? To enable patients to follow medical advice, we need to invest more in sensitizing women and their families about GDM and to tailor our interventions and recommendations to their needs and potential - not vice versa. Uniform GDM guidelines are an important first step but more needs to be done to avoid dissatisfaction on the part of both providers and clients. Otherwise the question asked by one respondent “*Why screen if we cannot follow-up and manage? Only to put pressure on the woman or on the care team?*” will not be satisfactorily addressed.

## Conclusion

Providers from LLMIC face various challenges related to screening for and management of GDM. Policy makers need to address these challenges by strengthening their respective health care system as a whole and by ensuring that non-communicable diseases are better integrated into existing packages of free or subsidized maternal health care.

## Abbreviations

ANC: Antenatal care
GDM: Gestational diabetes mellitus
HbA1C: Glycosylated hemoglobin
LLMIC: Low and lower middle income countries
OGTT: Oral glucose tolerance test
SMBG: Self-monitoring of blood glucose
